# Old Age

**Published:** 1879-03

**Authors:** 


Statistics of Old Age.—The census of 1854 shows us that the oldest person then living in the United States was 140. This person was an Indian woman, residing in North Carolina. In the same State was an Indian aged 125; a negro woman, 111; two black slaves, 110 each; one mulatto male, 120; and several white males and females from 106 to 114. In the parish of Lafayette, La., was a female, black, aged 120. In several of the States there were found persons, white and black, aged from 110 to 115. There were in the United States, in 1850, 2,555 persons over 100 years. This shows that about one person in 9,000 will be likely to live to that age.

